# Supplementary material for: Human and conservation factors affect spatial variation of reef fish assemblages in Colombian Pacific reefs
Source: PeerJ. 2025 Jun 18;13:e19482. doi: 10.7717/peerj.19482 (PMC12182057; doi:10.7717/peerj.19482)
Supplement: Supplemental Information 1 — Geographical position, number of fishermen, market distance, protection status, and underwater visual census information from locations sampled along the Colombian Pacific Coast. Protection status abbreviation: National Natural Park (PNN), Flora and Fauna Sanctuary (FFS), and Regional District of Integrated Management (DRMI, Spanish abbreviation). Number of total fishermen was extracted from national reports (Zuluaga et al., 2009; Cobos-Otálora et al., 2012; Velandia & Diaz, 2016). Protection age was extracted from Guzman et al. (2023). [file peerj-13-19482-s001.docx]

**Table S1. Location information.** Geographical position, number of fishermen, market distance, protection status, and underwater visual census information from locations sampled along the Colombian Pacific Coast. Protection status abbreviation: National Natural Park (PNN), Flora and Fauna Sanctuary (FFS), and Regional District of Integrated Management (DRMI, Spanish abbreviation). Number of total fishermen was extracted from national reports (Zuluaga et al. 2009, Cobos-Otálora et al. 2012, Velandia & Diaz 2016). Protection age was extracted from Guzman et al. (2023).

| **Location** | **Site** | **Coordinates**  (Lat and Log) | **Protection age (years)** | **Number of fishermen** | **Market distance (km)** | **Protection status** | **Transect area (m^2^)** | **Number of transects** | **Sampling year** | **Season** |
| --- | --- | --- | --- | --- | --- | --- | --- | --- | --- | --- |
| Bahía Solano | Piedra de Zapata | [6.369; -77.435] | 0 | 1305 | 16 | Not | 50 & 100 | 14 | 2015, 2016 | Rainy |
|  | Piedra del Norte | [6.316; -77.477] | 0 | 1305 | 13 | Not | 50 & 100 | 16 | 2015, 2016 | Rainy |
| Cabo Corrientes | Amargal | [5.579; -77.514] | 10 | 1398 | 31.5 | DRMI | 50 & 100 | 9 | 2015, 2016 | Rainy |
|  | Parguera | [5.609; -77.504] | 10 | 1398 | 28.4 | DRMI | 50 & 100 | 15 | 2015, 2016 | Rainy |
|  | Piedra Bonita | [5.594; -77.503] | 10 | 1398 | 29.5 | DRMI | 50 & 100 | 15 | 2015, 2016 | Rainy |
|  | Piedra Colo | [5.503; -77.533] | 10 | 1398 | 40 | DRMI | 50 & 100 | 13 | 2015, 2016 | Rainy |
|  | Piedra Oswaldo | [5.544; -77.515] | 10 | 1398 | 35.2 | DRMI | 50 & 100 | 10 | 2015, 2016 | Rainy |
|  | Punta Arusí | [5.611; -77.485] | 10 | 1398 | 26.6 | DRMI | 50 & 100 | 16 | 2015, 2016 | Rainy |
|  | Roñosa | [5.585; -77.514] | 10 | 1398 | 31 | DRMI | 50 & 100 | 17 | 2015, 2016 | Rainy |
| Cabo Marzo | La Foca | [6.785; -77.693] | 0 | 32 | 37.7 | Not | 50 & 100 | 9 | 2015, 2016 | Rainy |
|  | Piedra Eroito | [6.814; -77.692] | 0 | 32 | 32.6 | Not | 50 & 100 | 14 | 2015, 2016 | Rainy |
|  | Piedra Rodrigo | [6.783; -77.693] | 0 | 32 | 37.84 | Not | 50 & 100 | 17 | 2015, 2016 | Rainy |
|  | Punta faro | [6.824; -77.689] | 0 | 32 | 32 | Not | 50 | 9 | 2016 | Rainy |
| Cupica | Chicocora | [6.679; -77.427] | 0 | 32 | 51.7 | Not | 50 & 100 | 8 | 2016 | Rainy |
|  | La Mina | [6.686; -77.549] | 0 | 32 | 54.3 | Not | 50 | 10 | 2015, 2016 | Rainy |
|  | La Viuda | [6.633; -77.499] | 0 | 32 | 46.9 | Not | 50 & 100 | 6 | 2016 | Rainy |
|  | Parguera | [6.691; -77.541] | 0 | 32 | 54.6 | Not | 100 | 15 | 2015, 2016 | Rainy |
|  | Piedra Lalo | [6.644; -77.524] | 0 | 32 | 59.6 | Not | 50 & 100 | 8 | 2015 | Rainy |
| Golfo de Tribugá | Morro Mico | [5.871; -77.310] | 10 | 1398 | 18.9 | PNN | 50 & 100 | 14 | 2015, 2016 | Rainy |
|  | Morros Jurubidá | [5.813; -77.297] | 10 | 1398 | 12.2 | DRMI | 50 & 100 | 17 | 2015, 2016 | Rainy |
|  | Punta Orión | [5.930; -77.355] | 10 | 1398 | 26.7 | DRMI | 60 | 18 | 2015, 2016 | Rainy |
| Gorgona | La Azufrada | [2.950; -78.178] | 39 | 0 | 59 | PNN | 60 | 46 | 2006, 2009 | Rainy |
|  | El Laberinto | [3.004; -78.168] | 39 | 0 | 63 | PNN | 60 | 10 | 2006, 2009 | Rainy |
|  | El muelle | [2.961; -78.174] | 39 | 0 | 59.7 | PNN | 40 | 14 | 2016 | Rainy |
| Malpelo | El Arrecife | [4.004; -81.604] | 29 | 0 | 506 | FFS | 40 | 7 | 2015 | Rainy |
|  | Bajo del Junior | [3.998; -81.612] | 29 | 0 | 507 | FFS | 40 | 15 | 2015 | Rainy |
|  | La Nevera | [4.002; -81.611] | 29 | 0 | 508 | FFS | 40 | 16 | 2015 | Rainy |
|  | La Pared del náufrago | [4.007; -81.606] | 29 | 0 | 506 | FFS | 40 | 15 | 2015 | Rainy |

**References**

Zuluaga JSantiago, Díaz JManuel, Vieira Carlos, Moreno Evelyn, Mena Zulia, García Cándida, Montaño ANely, Mateus Rubén (2009) Diagnóstico integrado de la Unidad Ambiental Costera Pacífico Norte Chocoano.

Cobos-Otálora A, Galvis-Claro N, Fahrenberger AD, Granados-Diaz MC, Dueñas JD, Pardo-Galeano J, Gutiérrez-Gonzáles GG, Guillot LI, Muñoz-Lasso O, Neira A, Castaño-Vargas CA, Diaz-Velandia MC, Betancourt-Vieira CA (2012) La pesca artesanal en el norte del Pacífico colombiano. Fundación MarViva. Bogotá.

Guzman DH, Mier RL, Vergara A, Milanes CB (2023) Marine protected areas in Colombia: A historical review of legal marine protected since the late 1960 s to 2023. Marine Policy 155:105726.

Velandia MC, Diaz JM (2016) Atlas Marino-Costero del Pacifico Norte Colombiano. Fundación MarViva. 130.
