# Supplementary material for: Human and conservation factors affect spatial variation of reef fish assemblages in Colombian Pacific reefs
Source: PeerJ. 2025 Jun 18;13:e19482. doi: 10.7717/peerj.19482 (PMC12182057; doi:10.7717/peerj.19482)
Supplement: Supplemental Information 2 — Bold values indicate p < 0.005. [file peerj-13-19482-s002.docx]

Table S2. Output GLM evaluating whether fish density and biomass vary among locations along the Colombian Pacific Coast. Bold values indicate p <0.005.

| Location | Fish density | Fish biomass |
| --- | --- | --- |
| Cabo Corrientes | 0.351 [0.297, 0.405] | 0.091 [0.010, 0.171] |
|  | t-value=12.82; p-value<**0.001** | t-value=2.22; p-value=**0.026** |
| Cabo Marzo | 0.355 [0.296, 0.414] | 0.497 [0.408, 0.586] |
|  | t-value=11.80; p-value<**0.001** | t-value=10.99; p-value<**0.001** |
| Cupica | -0.142 [-0.198, -0.086] | 0.142 [0.057, 0.226] |
|  | t-value=-4.94; p-value<**0.001** | t-value=3.29; p-value<**0.001** |
| Golfo de Tribugá | -0.228 [-0.284, -0.172] | -0.421 [-0.506, -0.337] |
|  | t-value=-7.93; p-value<**0.001** | t-value=-9.766; p-value<**0.001** |
| Gorgona | -0.030 [-0.083, 0.024] | -0.367 [-0.447, -0.287] |
|  | t-value=-1.10; p-value=0.277 | t-value=-8.98; p-value<**0.001** |
| Malpelo | 0.890 [0.833, 0.946] | 1.311 [1.225, 1.395] |
|  | t-value=30.80; p-value<**0.001** | t-value=30.20; p-value<**0.001** |
