# Supplementary material for: Human and conservation factors affect spatial variation of reef fish assemblages in Colombian Pacific reefs
Source: PeerJ. 2025 Jun 18;13:e19482. doi: 10.7717/peerj.19482 (PMC12182057; doi:10.7717/peerj.19482)
Supplement: Supplemental Information 3 — Results of Similarity Percentage Analysis (SIMPER). [file peerj-13-19482-s003.docx]

**Table S3. Results of Similarity Percentage Analysis (SIMPER).** List of species that contribute to 70% density dissimilarity observed locations across the Colombian Pacific Coast.

| **Pairwise comparison (Location)** | **species** | **Global mean** | **SD** | **ratio** | **CumSum** | **p** |
| --- | --- | --- | --- | --- | --- | --- |
| Bahia Solano ⎯ Cabo Marzo | *Canthidermis maculata* | 0.059 | 0.068 | 0.876 | 0.563 | 0.034 |
| Bahia Solano ⎯ Cabo Marzo | *Decapterus macarellus* | 0.043 | 0.043 | 1.026 | 0.671 | 0.033 |
| Bahia Solano ⎯ Cabo Marzo | *Kyphosus ocyurus* | 0.078 | 0.071 | 1.119 | 0.512 | 0.039 |
| Bahia Solano ⎯ Cupica | *Acanthurus xanthopterus* | 0.020 | 0.020 | 1.017 | 0.663 | 0.007 |
| Bahia Solano ⎯ Cupica | *Canthidermis maculata* | 0.038 | 0.043 | 0.873 | 0.585 | 0.005 |
| Bahia Solano ⎯ Cupica | *Decapterus macarellus* | 0.053 | 0.058 | 0.914 | 0.565 | 0.005 |
| Bahia Solano ⎯ Golfo de Tribugá | *Acanthurus xanthopterus* | 0.019 | 0.019 | 1.065 | 0.698 | 0.024 |
| Bahia Solano ⎯ Golfo de Tribugá | *Canthidermis maculata* | 0.041 | 0.050 | 0.825 | 0.587 | 0.015 |
| Bahia Solano ⎯ Golfo de Tribugá | *Decapterus macarellus* | 0.049 | 0.058 | 0.849 | 0.563 | 0.017 |
| Bahia Solano ⎯ Golfo de Tribugá | *Kyphosus vaigiensis* | 0.018 | 0.009 | 2.334 | 0.692 | 0.004 |
| Bahia Solano ⎯ Golfo de Tribugá | *Lutjanus guttatus* | 0.020 | 0.023 | 0.912 | 0.691 | 0.045 |
| Bahia Solano ⎯ Golfo de Tribugá | *Stegastes flavilatus* | 0.021 | 0.016 | 1.451 | 0.676 | 0.030 |
| Bahia Solano ⎯ Gorgona | *Canthidermis maculata* | 0.047 | 0.055 | 0.865 | 0.529 | 0.031 |
| Bahia Solano ⎯ Gorgona | *Decapterus macarellus* | 0.044 | 0.050 | 0.882 | 0.605 | 0.034 |
| Bahia Solano ⎯ Gorgona | *Myripristis leiognathus* | 0.025 | 0.022 | 1.310 | 0.697 | 0.016 |
| Bahia Solano ⎯ Malpelo | *Apogon atradorsatus* | 0.212 | 0.125 | 1.717 | 0.280 | 0.008 |
| Bahia Solano ⎯ Malpelo | *Cirrhitichthys oxycephalus* | 0.081 | 0.041 | 2.337 | 0.615 | 0.024 |
| Cabo Corrientes ⎯ Cabo Marzo | *Kyphosus ocyurus* | 0.057 | 0.053 | 1.097 | 0.542 | 0.019 |
| Cabo Corrientes ⎯ Cupica | *Caranx caballus* | 0.017 | 0.019 | 0.904 | 0.694 | 0.043 |
| Cabo Corrientes ⎯ Cupica | *Chromis atrilobata* | 0.154 | 0.110 | 1.410 | 0.229 | 0.049 |
| Cabo Corrientes ⎯ Malpelo | *Apogon atradorsatus* | 0.180 | 0.104 | 1.744 | 0.236 | 0.002 |
| Cabo Corrientes ⎯ Malpelo | *Cirrhitichthys oxycephalus* | 0.067 | 0.035 | 2.081 | 0.568 | 0.010 |
| Cabo Marzo ⎯ Cupica | *Kyphosus ocyurus* | 0.071 | 0.065 | 1.106 | 0.469 | 0.003 |
| Cabo Marzo ⎯ Golfo de Tribugá | *Kyphosus ocyurus* | 0.070 | 0.067 | 1.056 | 0.463 | 0.019 |
| Cabo Marzo ⎯ Gorgona | *Kyphosus ocyurus* | 0.064 | 0.060 | 1.100 | 0.447 | 0.030 |
| Cabo Marzo ⎯ Malpelo | *Apogon atradorsatus* | 0.189 | 0.111 | 1.716 | 0.256 | 0.002 |
| Cabo Marzo ⎯ Malpelo | *Cirrhitichthys oxycephalus* | 0.071 | 0.038 | 2.086 | 0.538 | 0.011 |
| Cupica ⎯ Golfo de Tribugá | *Abudefduf troschelii* | 0.023 | 0.016 | 1.391 | 0.600 | 0.045 |
| Cupica ⎯ Golfo de Tribugá | *Kyphosus vaigiensis* | 0.016 | 0.010 | 1.737 | 0.688 | 0.002 |
| Cupica ⎯ Golfo de Tribugá | *Lutjanus guttatus* | 0.019 | 0.022 | 0.887 | 0.658 | 0.009 |
| Cupica ⎯ Gorgona | *Acanthurus triostegus* | 0.017 | 0.026 | 0.659 | 0.661 | 0.006 |
| Cupica ⎯ Gorgona | *Hemiramphus saltator* | 0.017 | 0.026 | 0.664 | 0.675 | 0.005 |
| Cupica ⎯ Gorgona | *Heteroconger klausewitzi* | 0.040 | 0.060 | 0.664 | 0.485 | 0.005 |
| Cupica ⎯ Gorgona | *Myripristis leiognathus* | 0.023 | 0.022 | 1.097 | 0.612 | 0.002 |
| Cupica ⎯ Malpelo | *Apogon atradorsatus* | 0.211 | 0.122 | 1.749 | 0.270 | 0.001 |
| Cupica ⎯ Malpelo | *Cirrhitichthys oxycephalus* | 0.080 | 0.041 | 2.208 | 0.595 | 0.004 |
| Cupica ⎯ Malpelo | *Paranthias colonus* | 0.170 | 0.058 | 3.078 | 0.448 | 0.034 |
| Golfo de Tribugá ⎯ Gorgona | *Acanthurus triostegus* | 0.017 | 0.026 | 0.636 | 0.690 | 0.038 |
| Golfo de Tribugá ⎯ Gorgona | *Heteroconger klausewitzi* | 0.038 | 0.059 | 0.643 | 0.492 | 0.045 |
| Golfo de Tribugá ⎯ Gorgona | *Kyphosus vaigiensis* | 0.017 | 0.007 | 2.623 | 0.686 | 0.003 |
| Golfo de Tribugá ⎯ Gorgona | *Myripristis leiognathus* | 0.021 | 0.022 | 1.042 | 0.639 | 0.018 |
| Golfo de Tribugá ⎯ Gorgona | *Stegastes flavilatus* | 0.020 | 0.011 | 1.927 | 0.642 | 0.020 |
| Golfo de Tribugá ⎯ Malpelo | *Apogon atradorsatus* | 0.204 | 0.120 | 1.704 | 0.259 | 0.002 |
| Golfo de Tribugá ⎯ Malpelo | *Cirrhitichthys oxycephalus* | 0.077 | 0.041 | 2.119 | 0.555 | 0.012 |
| Gorgona ⎯ Malpelo | *Apogon atradorsatus* | 0.194 | 0.114 | 1.714 | 0.260 | 0.004 |
| Gorgona ⎯ Malpelo | *Cirrhitichthys oxycephalus* | 0.061 | 0.038 | 1.613 | 0.608 | 0.045 |
