# Supplementary material for: Human and conservation factors affect spatial variation of reef fish assemblages in Colombian Pacific reefs
Source: PeerJ. 2025 Jun 18;13:e19482. doi: 10.7717/peerj.19482 (PMC12182057; doi:10.7717/peerj.19482)
Supplement: Supplemental Information 4 — Results of Similarity Percentage Analysis (SIMPER). [file peerj-13-19482-s004.docx]

**Table S4. Results of Similarity Percentage Analysis (SIMPER).** List of species that contribute to 70% biomass dissimilarity observed locations across the Colombian Pacific Coast.

| **Pairwise comparison (Location)** | **Species** | **Global mean** | **SD** | **ratio** | **CumSum** | **p** |
| --- | --- | --- | --- | --- | --- | --- |
| Bahia Solano ⎯ Cabo Corrientes | *Canthidermis maculata* | 0.171 | 0.190 | 0.880 | 0.277 | 0.042 |
| Bahia Solano ⎯ Cabo Corrientes | *Decapterus macarellus* | 0.049 | 0.061 | 0.796 | 0.540 | 0.037 |
| Bahia Solano ⎯ Cabo Corrientes | *Scarus ghobban* | 0.021 | 0.025 | 0.846 | 0.677 | 0.042 |
| Bahia Solano ⎯ Cabo Corrientes | *Scomberomorus sierra* | 0.024 | 0.030 | 0.775 | 0.679 | 0.040 |
| Bahia Solano ⎯ Cupica | *Canthidermis maculata* | 0.199 | 0.219 | 0.895 | 0.285 | 0.012 |
| Bahia Solano ⎯ Cupica | *Decapterus macarellus* | 0.062 | 0.071 | 0.865 | 0.432 | 0.007 |
| Bahia Solano ⎯ Cupica | *Scarus ghobban* | 0.022 | 0.023 | 1.049 | 0.673 | 0.017 |
| Bahia Solano ⎯ Cupica | *Scomberomorus sierra* | 0.031 | 0.036 | 0.847 | 0.579 | 0.005 |
| Bahia Solano ⎯ Cupica | *Sufflamen verres* | 0.021 | 0.021 | 1.018 | 0.683 | 0.030 |
| Bahia Solano ⎯ Golfo de Tribugá | *Abudefduf troschelii* | 0.024 | 0.014 | 2.211 | 0.669 | 0.048 |
| Bahia Solano ⎯ Golfo de Tribugá | *Arothron meleagris* | 0.024 | 0.021 | 1.250 | 0.614 | 0.022 |
| Bahia Solano ⎯ Golfo de Tribugá | *Canthidermis maculata* | 0.198 | 0.223 | 0.876 | 0.298 | 0.019 |
| Bahia Solano ⎯ Golfo de Tribugá | *Decapterus macarellus* | 0.061 | 0.071 | 0.852 | 0.511 | 0.016 |
| Bahia Solano ⎯ Golfo de Tribugá | *Scarus ghobban* | 0.023 | 0.020 | 1.302 | 0.680 | 0.030 |
| Bahia Solano ⎯ Golfo de Tribugá | *Scomberomorus sierra* | 0.030 | 0.035 | 0.845 | 0.603 | 0.016 |
| Bahia Solano ⎯ Golfo de Tribugá | *Sufflamen verres* | 0.024 | 0.025 | 0.980 | 0.673 | 0.025 |
| Bahia Solano ⎯ Gorgona | *Canthidermis maculata* | 0.191 | 0.215 | 0.878 | 0.284 | 0.026 |
| Bahia Solano ⎯ Gorgona | *Decapterus macarellus* | 0.055 | 0.064 | 0.865 | 0.476 | 0.028 |
| Bahia Solano ⎯ Gorgona | *Scomberomorus sierra* | 0.028 | 0.033 | 0.842 | 0.656 | 0.027 |
| Bahia Solano ⎯ Gorgona | *Sufflamen verres* | 0.024 | 0.026 | 0.948 | 0.698 | 0.030 |
| Bahia Solano ⎯ Malpelo | *Dermatolepis dermatolepis* | 0.042 | 0.032 | 1.318 | 0.662 | 0.032 |
| Bahia Solano ⎯ Malpelo | *Gymnothorax dovii* | 0.042 | 0.027 | 1.599 | 0.656 | 0.016 |
| Cabo Corrientes ⎯ Cabo Marzo | *Prionurus laticlavius* | 0.050 | 0.047 | 1.089 | 0.512 | 0.007 |
| Cabo Corrientes ⎯ Golfo de Tribugá | *Lutjanus guttatus* | 0.021 | 0.022 | 0.963 | 0.672 | 0.037 |
| Cabo Corrientes ⎯ Gorgona | *Gymnothorax flavimarginatus* | 0.043 | 0.066 | 0.666 | 0.505 | 0.038 |
| Cabo Corrientes ⎯ Malpelo | *Dermatolepis dermatolepis* | 0.036 | 0.029 | 1.279 | 0.616 | 0.004 |
| Cabo Corrientes ⎯ Malpelo | *Gymnothorax dovii* | 0.037 | 0.025 | 1.549 | 0.612 | 0.002 |
| Cabo Corrientes ⎯ Malpelo | *Lutjanus jordani* | 0.095 | 0.116 | 0.784 | 0.305 | 0.039 |
| Cabo Corrientes ⎯ Malpelo | *Lutjanus viridis* | 0.064 | 0.077 | 0.883 | 0.439 | 0.030 |
| Cabo Marzo ⎯ Cupica | *Arothron hispidus* | 0.034 | 0.038 | 0.969 | 0.578 | 0.042 |
| Cabo Marzo ⎯ Cupica | *Gymnothorax castaneus* | 0.031 | 0.033 | 1.077 | 0.588 | 0.036 |
| Cabo Marzo ⎯ Cupica | *Prionurus laticlavius* | 0.057 | 0.052 | 1.110 | 0.406 | 0.003 |
| Cabo Marzo ⎯ Golfo de Tribugá | *Prionurus laticlavius* | 0.059 | 0.052 | 1.173 | 0.479 | 0.013 |
| Cabo Marzo ⎯ Gorgona | *Prionurus laticlavius* | 0.057 | 0.050 | 1.185 | 0.434 | 0.019 |
| Cabo Marzo ⎯ Malpelo | *Dermatolepis dermatolepis* | 0.035 | 0.027 | 1.282 | 0.595 | 0.009 |
| Cabo Marzo ⎯ Malpelo | *Gymnothorax dovii* | 0.035 | 0.021 | 1.718 | 0.586 | 0.004 |
| Cupica ⎯ Golfo de Tribugá | *Abudefduf troschelii* | 0.020 | 0.016 | 1.278 | 0.674 | 0.028 |
| Cupica ⎯ Golfo de Tribugá | *Anisotremus caesius* | 0.019 | 0.019 | 1.022 | 0.692 | 0.002 |
| Cupica ⎯ Golfo de Tribugá | *Arothron meleagris* | 0.019 | 0.020 | 0.983 | 0.695 | 0.021 |
| Cupica ⎯ Golfo de Tribugá | *Lutjanus guttatus* | 0.026 | 0.028 | 0.907 | 0.619 | 0.018 |
| Cupica ⎯ Golfo de Tribugá | *Scarus ghobban* | 0.019 | 0.025 | 0.771 | 0.693 | 0.021 |
| Cupica ⎯ Gorgona | *Acanthurus triostegus* | 0.022 | 0.035 | 0.636 | 0.657 | 0.011 |
| Cupica ⎯ Gorgona | *Gymnothorax castaneus* | 0.048 | 0.069 | 0.703 | 0.357 | 0.023 |
| Cupica ⎯ Gorgona | *Gymnothorax flavimarginatus* | 0.053 | 0.077 | 0.704 | 0.368 | 0.006 |
| Cupica ⎯ Gorgona | *Heteroconger klausewitzi* | 0.076 | 0.115 | 0.659 | 0.197 | 0.014 |
| Cupica ⎯ Gorgona | *Myripristis berndti* | 0.019 | 0.015 | 1.486 | 0.694 | 0.001 |
| Cupica ⎯ Gorgona | *Scarus perrico* | 0.106 | 0.155 | 0.682 | 0.139 | 0.018 |
| Cupica ⎯ Malpelo | *Caranx melampygus* | 0.022 | 0.029 | 0.779 | 0.679 | 0.036 |
| Cupica ⎯ Malpelo | *Cirrhitus rivulatus* | 0.019 | 0.023 | 0.848 | 0.699 | 0.015 |
| Cupica ⎯ Malpelo | *Dermatolepis dermatolepis* | 0.039 | 0.030 | 1.300 | 0.583 | 0.001 |
| Cupica ⎯ Malpelo | *Gymnothorax dovii* | 0.043 | 0.027 | 1.649 | 0.560 | 0.001 |
| Cupica ⎯ Malpelo | *Lutjanus jordani* | 0.106 | 0.127 | 0.796 | 0.299 | 0.008 |
| Cupica ⎯ Malpelo | *Lutjanus viridis* | 0.070 | 0.082 | 0.920 | 0.444 | 0.014 |
| Cupica ⎯ Malpelo | *Mycteroperca olfax* | 0.022 | 0.018 | 1.291 | 0.690 | 0.001 |
| Cupica ⎯ Malpelo | *Paranthias colonus* | 0.114 | 0.068 | 1.756 | 0.257 | 0.043 |
| Cupica ⎯ Malpelo | *Seriola rivoliana* | 0.101 | 0.138 | 0.747 | 0.308 | 0.042 |
| Golfo de Tribugá ⎯ Gorgona | *Acanthurus triostegus* | 0.022 | 0.035 | 0.632 | 0.651 | 0.045 |
| Golfo de Tribugá ⎯ Gorgona | *Gymnothorax flavimarginatus* | 0.052 | 0.076 | 0.704 | 0.447 | 0.031 |
| Golfo de Tribugá ⎯ Gorgona | *Heteroconger klausewitzi* | 0.076 | 0.117 | 0.650 | 0.291 | 0.047 |
| Golfo de Tribugá ⎯ Gorgona | *Myripristis berndti* | 0.019 | 0.014 | 1.526 | 0.696 | 0.005 |
| Golfo de Tribugá ⎯ Malpelo | *Dermatolepis dermatolepis* | 0.040 | 0.031 | 1.301 | 0.600 | 0.009 |
| Golfo de Tribugá ⎯ Malpelo | *Gymnothorax dovii* | 0.042 | 0.027 | 1.667 | 0.577 | 0.003 |
| Gorgona ⎯ Malpelo | *Dermatolepis dermatolepis* | 0.039 | 0.030 | 1.313 | 0.584 | 0.009 |
| Gorgona ⎯ Malpelo | *Gymnothorax dovii* | 0.041 | 0.025 | 1.690 | 0.572 | 0.004 |
| Gorgona ⎯ Malpelo | *Paranthias colonus* | 0.125 | 0.060 | 2.227 | 0.226 | 0.036 |
