# Supplementary material for: Human and conservation factors affect spatial variation of reef fish assemblages in Colombian Pacific reefs
Source: PeerJ. 2025 Jun 18;13:e19482. doi: 10.7717/peerj.19482 (PMC12182057; doi:10.7717/peerj.19482)
Supplement: Supplemental Information 5 — Effects of human and conservation factors on species richness, density, and biomass of fish assemblages observed along the Colombian Pacific Coast. Bold values indicate p < 0.005. [file peerj-13-19482-s005.docx]

Table S5. Results of Linear Mixed Models, excluding Malpelo Island. Effects of human and conservation factors on species richness, density, and biomass of fish assemblages observed along the Colombian Pacific Coast. Bold values indicate p <0.005.

|  | Species richness | Fish density | Fish biomass |
| --- | --- | --- | --- |
| Number of fishermen | -0.006 [-0.097, 0.086] | 0.262 [-0.208, 0.732] | 0.227 [-0.516, 0.969] |
|  | t-value=-0.129 | t-value=1.173 | t-value=0.641 |
|  | p-value=0.899 | p-value=0.256 | p-value=0.530 |
| Market distance | -0.023 [-0.205, 0.158] | 0.460 [-0.296, 1.215] | 0.429 [-0.955, 1.813] |
|  | t-value=-0.267 | t-value=1.278 | t-value=0.652 |
|  | p-value=0.792 | p-value=0.217 | p-value=0.523 |
| Protection status | 0.032 [-0.026, 0.090] | 0.107 [-0.189, 0.403] | -0.084 [-0.544, 0.376] |
|  | t-value=1.166 | t-value=0.759 | t-value=-0.384 |
|  | p-value=0.259 | p-value=0.458 | p-value=0.706 |
| Num.Obs. | 24 | 24 | 24 |
