# Supplementary material for: Human and conservation factors affect spatial variation of reef fish assemblages in Colombian Pacific reefs
Source: PeerJ. 2025 Jun 18;13:e19482. doi: 10.7717/peerj.19482 (PMC12182057; doi:10.7717/peerj.19482)
Supplement: Supplemental Information 6 — Effects of human factors on species richness, density, and biomass of fish assemblages observed along the Colombian Pacific Coast. Bold values indicate p < 0.005. [file peerj-13-19482-s006.docx]

Table S6. Results of PERMANOVA tests, excluding Malpelo. Effects of human factors on species richness, density, and biomass of fish assemblages observed along the Colombian Pacific Coast. Bold values indicate p <0.005.

| **Fish metric** | **Factors** | **df** | **R2** | **F-value** | **p-value** |
| --- | --- | --- | --- | --- | --- |
| Species richness | Number of fishermen | 1 | 0.01 | 1.38 | 0.27 |
|  | Market distance | 1 | 0.01 | 1.48 | 0.23 |
|  | Protection status | 1 | 0.01 | 1.32 | 0.28 |
|  | Residuals | 20 | 0.16 |  |  |
|  |  |  |  |  |  |
| Fish density | Number of fishermen | 1 | 0.08 | 1.30 | 0.26 |
|  | Market distance | 1 | 0.05 | 0.80 | 0.46 |
|  | Protection status | 1 | 0.04 | 0.73 | 0.52 |
|  | Residuals | 20 | 1.29 |  |  |
|  |  |  |  |  |  |
| Fish biomass | Number of fishermen | 1 | 0.14 | 0.94 | 0.42 |
|  | Market distance | 1 | 0.25 | 1.64 | **0.04** |
|  | Protection status | 1 | 0.04 | 0.28 | 0.94 |
|  | Residuals | 20 | 3.10 |  |  |
